# Recent and Upcoming Drug Therapies for Pediatric Heart Failure

**DOI:** 10.3389/fped.2021.681224

**Published:** 2021-11-11

**Authors:** Karla L. Loss, Robert E. Shaddy, Paul F. Kantor

**Affiliations:** Division of Cardiology, Department of Pediatrics, Keck School of Medicine at University of Southern California, Children's Hospital Los Angeles, Los Angeles, CA, United States

**Keywords:** pediatric heart failure, sacubitril/valsartan, ivabradine, omecamtiv mecarbil, heart failure reduced ejection fraction (HFrEF)

## Abstract

Pediatric heart failure (HF) is an important clinical condition with high morbidity, mortality, and costs. Due to the heterogeneity in clinical presentation and etiologies, the development of therapeutic strategies is more challenging in children than adults. Most guidelines recommending drug therapy for pediatric HF are extrapolated from studies in adults. Unfortunately, even using all available treatment, progression to cardiac transplantation is common. The development of prospective clinical trials in the pediatric population has significant obstacles, including small sample sizes, slow recruitment rates, challenging endpoints, and high costs. However, progress is being made as evidenced by the recent introduction of ivabradine and of sacubitril/valsartan. In the last 5 years, new drugs have also been developed for HF with reduced ejection fraction (HFrEF) in adults. The use of well-designed prospective clinical trials will be fundamental in the evaluation of safety and efficacy of these new drugs on the pediatric population. The aim of this article is to review the clinical presentation and management of acute and chronic pediatric heart failure, focusing on systolic dysfunction in patients with biventricular circulation and a systemic left ventricle. We discuss the drugs recently approved for children and those emerging, or in use for adults with HFrEF.

## Introduction and Classification of Heart Failure

Pediatric heart failure (HF) can be defined as a clinical syndrome resulting from ventricular dysfunction, and volume or pressure overload, alone or in combination (1, 2). The two most common pathophysiological categories resulting in end stage HF in children are cardiomyopathy and congenital heart disease (CHD), each contributing about half of the cases resulting in cardiac transplant, according to recent International Society for Heart and Lung Transplant data (3). Cardiomyopathy presents with predictable phenotypes, and dilated cardiomyopathy (DCM) or LV non-compaction cardiomyopathy (LVNC) typically manifest clinically as HF with reduced ejection fraction (HFrEF): this term implies by convention, the presence of symptomatic HF, a dilated left ventricle and an LV ejection fraction (EF) of <50%. Heart failure due to cardiomyopathy can manifest clinically at any age, including in the fetus, and in the case of DCM shows a peak incidence in the 1st year of life. Both the Pediatric Cardiomyopathy Registry in the USA and a national population survey in Australia suggested a similar population prevalence of 1.2/100,000 with a median age of presentation of 1.8 years (2, 4). The etiology of DCM is diverse, with about 1/3 of patients having an identified genetic mutation of one of a variety of sarcomeric proteins, resulting in a dilated ventricle, with eccentric hypertrophy, and poor systolic function. Over 35 such pathogenic mutations are now recognized, with inherited metabolic diseases and neuromuscular disorders also noted as etiologies (5–7). Acquired causes of DCM include acute viral myocarditis, cardiotoxicity following anthracycline exposure, nutritional (including trace element, vitamin, and iron) deficiency states, and Kawasaki disease (7).

The etiologies of congenital heart disease associated with HF in children are also well-described: there is added complexity in this situation however, since the patient may have a single or biventricular “circulation,” and the systemic ventricle may be a morphologic left or right ventricle. In general terms the “rules” of management for these situations cannot be directly extrapolated from that of a biventricular heart with a systemic left ventricle. Furthermore, in HF with a biventricular heart and a systemic left ventricle, although LV dilation is common, EF may actually be preserved: this is usually the case with left to right shunt lesions, such as a persistent patent arterial duct, a ventricular septal defect. Here, the presentation that is recognized as HF syndrome in infants and young children is commonly associated with a normal LV EF.

Additionally, there is some evidence that the genetic architecture of congenital heart disease (which is polygenic) overlaps with that of cardiomyopathy (generally considered to be monogenic) (8). Certainly there are associations with cardiomyopathy in some well-recognized genetic syndromes, 1p36- being a well-recognized example. Left ventricular non-compaction cardiomyopathy is also well-described to coexist with several congenital heart disease entities.

Although well-recognized, and an important acute and chronic disorder worldwide, the global incidence and prevalence of pediatric HF is unclear (9, 10). In most developed countries, over 70% of hemodynamically significant congenital heart disease has antenatal or postnatal diagnosis before hospital discharge, a figure greatly assisted by the universal initiation of newborn oximetry screening (11). In a systematic review, researchers described a wide range of incidence of pediatric HF varying from 0.87 (UK and Ireland) to 83.3 (Spain) per 100,000 (7). Although the number of patients with pediatric HF appears to be relatively small in comparison with adults suffering from HF in developed countries, the duration of hospitalization, mortality rates and costs are proportionally much higher (12).

Given all of this complexity, management of HF in children can be challenging, due to limited guideline availability and considerable practice variation. We will briefly review the clinical presentation and pharmacologic management of acute decompensated and chronic pediatric HF. We will emphasize recently approved therapies, and those still on the horizon for children, which are emerging or currently in use for adults with HFrEF. We will limit the scope of this review to left ventricular failure in children with biventricular physiology, and a systemic left ventricle, since there are very few studies, and no compelling evidence to support the use of chronic heart failure therapies for patients with the Fontan circulation, or with a systemic morphologic right ventricle.

## Acute Decompensated Heart Failure

**Figure 1:**
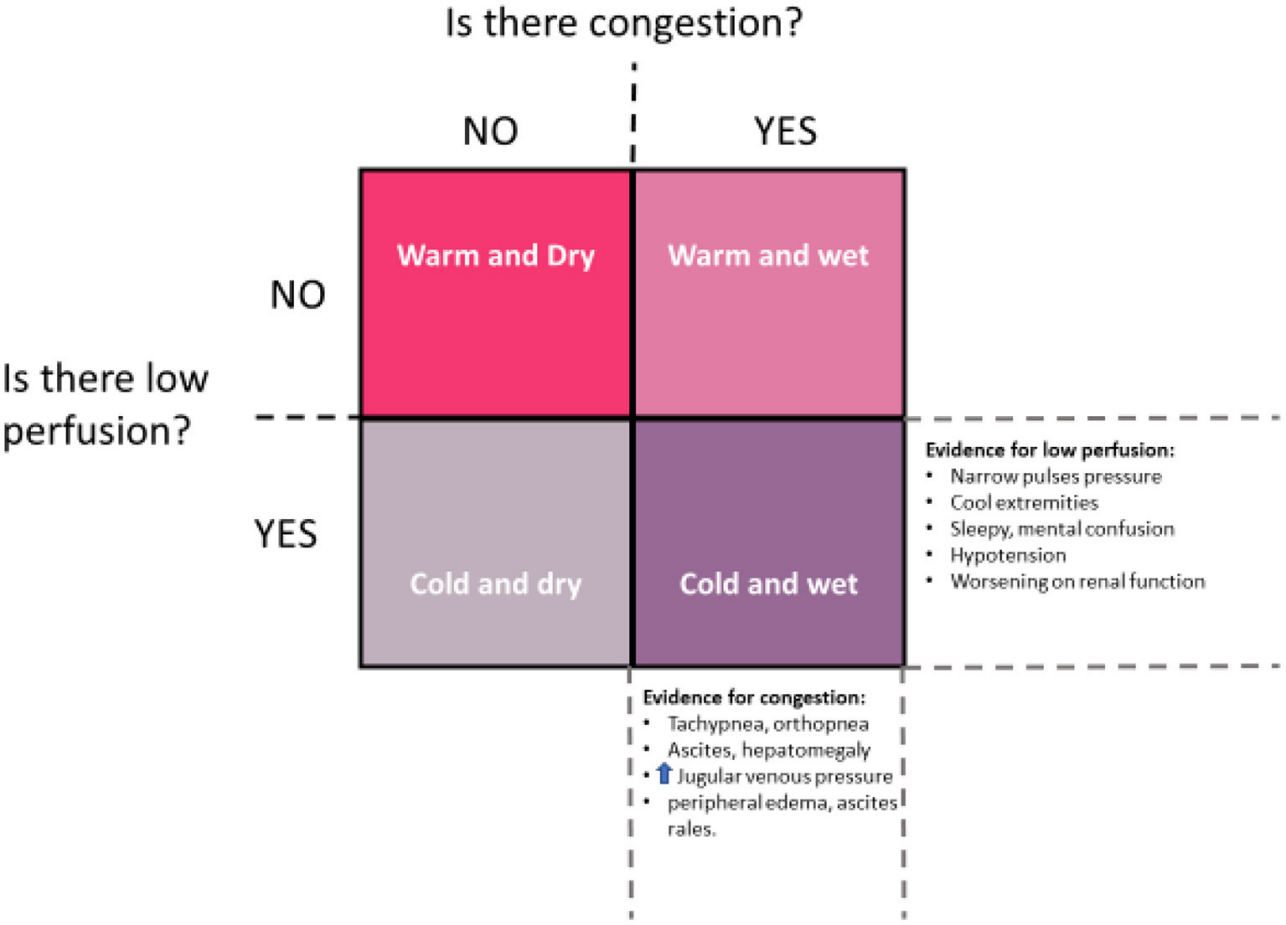
Clinical assessment in ADHF should answer the two questions suggested by this diagram: First, does the patient present with significant congestion? Second, does the patient present with significant underperfusion? Using this construct, patients will segregate into one of four categories in accordance with clinical findings. Typically, patients move in a clockwise fashion through these categories, first becoming congested (warm and wet) and then vasoconstricted to maintain blood pressure (cold and wet). Once vasoactive support and diuresis is achieved, movement is generally counter-clockwise, from cold and wet, to warm and wet, and then warm and dry. However, some patients will remain underperfused despite restoration of normovolemia, representing the cold and dry group, for whom mechanical support may be needed [image redrawn from Kantor and Mertens (13)].

As noted, in the young child, feeding difficulties, growth failure, irritability, and respiratory distress are the classical presenting symptoms of HF, while in older patients fatigue and exercise intolerance are more prevalent. Acute decompensated heart failure (ADHF) is characterized by an abrupt presentation, either *de novo* or as a deterioration of pre-existing symptoms, commonly with no identifiable precipitating event or illness. Patients demonstrate some degree of fluid retention (congestion) which can be offset by vomiting and poor feeding in infants and young children. Most will also have poor perfusion, permitting the broad categorization of patients into a simplified matrix of congestion/no congestion and underperfusion/no underperfusion (Figure 1).

The presence of congestion and/or hypo-perfusion, and whether the left or right ventricle are both involved in the dysfunction will also influence the signs and symptoms presented by the patient with pediatric HF. Right ventricular HF will generate jugular venous distention, hepatomegaly, and occasionally peripheral edema and ascites; on the other hand, left ventricular HF presents more frequently as dyspnea reflecting elevated left atrial pressure, with effort and feeding intolerance, pallor, abdominal pain, or syncope (14).

The presence of ADHF at initial presentation remains an important risk factor for eventual death or cardiac transplantation in children, despite improved outcomes in the contemporary era (15, 16). Therefore a high priority is placed on early recognition of this syndrome (13). Accurate diagnosis depends on a careful clinical history and physical examination. A chest x-ray, electrocardiogram, selected laboratory tests including brain natriuretic peptide (BNP) or its cleavage product amino-terminal pro brain natriuretic peptide (NT-proBNP) and a transthoracic echocardiogram will add fundamental information regarding the etiology and severity of HF. A comprehensive diagnostic workup searching for underlying causes is essential in patients with cardiomyopathy (14). Imaging exams include cardiac magnetic resonance imaging, computed tomography and catheterization, are occasionally indicated for complex cases of CHD. Above all it is necessary for these patients to be treated by a center with expertise in critical care and pediatric cardiology, since this is a high risk, high mortality condition.

The immediate treatment of ADHF is simultaneously a process of reversing hemodynamic instability, and of discovering potential reversible causes as diverse as inherited tachyarrhythmia, acquired myocarditis or congenital anomalous origin of the left coronary artery from the pulmonary artery in infants. Treatment is directed at remedying any reversible cause, and addressing the primary symptomatic profile of the patient (Figure 1). The medical therapies required in the treatment of ADHF in children are listed in Table 1 and comprise diuretics, vasoactive drugs and inotropic support, with or without mechanical ventilation. Loop diuretics (furosemide, bumetanide) remain first line therapy for these patients, while inotropic agents are indicated to restore perfusion pressure, to reverse end-organ failure and enable diuresis. Milrinone, dopamine and epinephrine are the most common vasoactive drugs used in clinical practice. There are no controlled clinical trials of these agents in children, except for the PRIMACORP trial of milrinone in post-operative congenital heart disease, but the use of these medications appears to at temporarily improve cardiac output and rescue end-organ perfusion in most pediatric patients with ADHF, due to HFrEF (18, 19).

**Table 1:** Medical therapies commonly required in the treatment of ADHF (17).

Currently used drugs for pediatric acute decompensated heart failure		
Diuretic	Furosemide	0.5–2 mg/kg q6–12 h0.1–0.4 mg/kg/h continuous infusion
	Bumetanide	0.015–0.1 mg/kg/dose every 6–24 h
	Clorothiazide	4–10 mg/kg/d divided every 12–24 h (maximum 20 mg/kg/d or 500 mg)
Vasoactive and Inotropic	Milrinone	0.25–1 μg/kg/min
	Dopamine	3–5 μg/kg/min
	Dobutamine	2.5–10 μg/kg/min
	Epinephrine	0.01–0.1 μg/kg/min
Vasodilator	Nitroprussite	0.3–4 μg/kg/min; maximum: 6 μg/kg/min for neonates, 12 μg/kg/min for children

Having re-established adequate perfusion and diuresis, the chronic use of vasoactive drugs is only indicated as a bridge to a mechanical support device or to heart transplantation (9). In patients who are stabilized, a transition to maintenance oral HF therapy is initiated as a means of preventing recurrence of ADHF, and managing the symptoms of chronic HF is the next step.

### Newer Drugs in Acute Heart Failure

#### Levosimendan

Levosimendan is licensed in over 60 countries, but not in the United States or Canada, since the FDA has not accepted that there is convincing evidence of efficacy from the published clinical trials. It is an intravenously administered vasoactive agent which increases cardiac contractility by calcium sensitization of troponin C, and reduces cardiac afterload by vasodilatation *via* the opening of the potassium channels on the sarcolemma of vascular smooth muscle; additionally, a cardioprotective effect has been postulated, due to the opening of mitochondrial potassium channels in cardiomyocytes by levosimendan (20–23). All these potential benefits are theoretically achieved without increasing myocardial oxygen consumption. However, there are features of this drug that are dissimilar pure calcium sensitizers, in that there is no prolongation of diastolic tension demonstrated *in vitro* with isolated muscle strips, perhaps due to the active metabolite of levosimendan, OR-1896, which governs many of the observed effects, and acts by inhibiting phosphodiesterase III, and reducing the breakdown of cAMP (24). Due to its mechanism of action, levosimendan has been studied to determine if it could be beneficial to patients with ADHF. However, event-driven clinical trials in adults with ADHF have been largely conducted in comparison to dobutamine, and even then, survival benefits have been unclear: the international phase III SURVIVE trial did not replicate the survival benefits suggested from earlier phase II studies, and the REVIVE trial demonstrated an increase in ventricular dysrhythmias (25, 26). The most prevalent side effect of levosimendan is hypotension, so the drug must be added cautiously to patients with low blood pressure, especially in the presence of possible hypovolemia (23). There are several smaller randomized clinical trials which have evaluated levosimendan following pediatric cardiac surgery, suggesting a benefit on hemodynamic parameters, but no effect on length of hospitalization or survival (27, 28). No randomized clinical studies have yet evaluated the efficacy of levosimendan in pediatric HFrEF, but retrospective reports describe the single or repeated infusion of levosimendan in pediatric patients with chronic severe systolic dysfunction due to DCM, and these are summarized in Table 2. Although some consider the evidence base promising, demonstrating improved cardiac output and end-organ function, the lack of control groups and small sample sizes in these studies is a limitation (35–38). A possible niche role for this agent may be in improving hemodynamic function in patients already on chronic β-blocker treatment, thereby avoiding classical inotropic agents if ADHF recurs. Levosimendan may also be helpful in patients with ADHF who are unresponsive to traditional inotropes (39). Another possible scenario for the use of levosimendan is in the palliative care of patients with end-stage HF where heart transplantation or long-term mechanical cardiac support is not achievable, given the reported benefit of reduced re-hospitalization rates in adults with advanced HF (40).

**Table 2:** Summary of previous published papers describing the effect of levosimendan on pediatric heart failure population.

**Author, year and study design**	**Study population**	**Sample size**	**Intervention and control group**	**Main results**
Namachivayam, 2006 (29) Case series	End stage or acute HF	15 patients	Single or repeated doses of levosimendan No control group.	Reduction on the dosage of dobutamine at day 5. Improvement of LV EF in the acute HF group. 1 patient had ventricular tachycardia. 4 patients died during the ICU hospitalization.
Ryerson, 2011 (30) Case Series	Severe decompensated HF, chronically dependent of IV vasoactive drugs	9 patients	Levosimendan rotation with dobutamine and/or milrinone No control group.	Helped to the discharge of ICU and wean of invasive mechanical ventilation. 2 deaths in hospital No improvement on LV ejection fraction
Prijić, 2011 (31) Case series	Severe decompensated HF with congenital or acquired heart disease chronically dependent of IV vasoactive drugs	3 patients	Levosimendan initiation and stop the previous vasoactive regimen No control group.	Clinical and echocardiography improvement with the improved EF and stroke volume. Reduction in heart rate in all the treated patients Normalization of lactate
Bravo, 2011 (32) Prospective, case series	Infants with CHD with low cardiac output syndrome refractory to conventional treatment	5 patients (7 doses of levosimendan)	Levosimendan (2 patients with repeated dosages)	Reduction on lactate and heart rate Improved the cerebral intravascular oxygenation (NIR-SRS parameters)
Suominen, 2011 (33) Single center, Retrospective descriptive data and survey data	3 groups: Cardiac surgery group (pre- peri or postoperative) Cardiac failure group (ADHF) Dilated cardiomyopathy group (acute or chronic HF)	293 patients (484 infusions)	Levosimendan (single or repeated infusions) No control group.	Descriptive data on use of levosimendan regarding gender distribution, median age, duration of infusion and interval between repeated dosages. For the efficacy and adverse events analysis, the results were based on the survey information. For 88.9% of the respondents levosimendan was considered as safe and efficacious The physicians were able to recall as adverse events: hypotension (62.1%), tachycardia (27.8%) occurred in the beginning of the infusion or no adverse events (27.8%).
Apostolopoulou, 2018 (34) Retrospective, single center.	End-stage pediatric HF or CHD refractory to treatment, functional class III or IV, chronically dependent of IV vasoactive and inotropic support.	27 patients	Long-term continuous intravenous ambulatory inotropic support (milrinone and dobutamine) and/or periodic levosimendan infusions as bridging to recovery, bridge to therapy or destination No control group.	Ambulatory inotropy – median time duration 1.0 (0.3–3.7) years Bridge to recovery: 6 patients with myocarditis, 4 with ambulatory inotropic + levosimendan and 2 with repeated infusions of levosimendan. All recovered. Bridge to heart transplant: 6 patients, 4 received ambulatory inotropic + levosimendan and 2 with repeated infusions of levosimendan. 3 deaths. Mainstain therapy: 15 patients, 1 received a VAD, 6 received ambulatory inotropic + levosimendan and 4 with repeated infusions of levosimendan. 4 deaths, median follow-up 2.1 (0.3–21.3) years. Adverse events: 4 central line infection or 4 central line dislodgements.

#### Serelaxin

Serelaxin is a recombinant form of a human relaxin-2 with vasodilator effect and direct effect on protection of end-organ function (41). This medication was initially investigated vs. placebo on the RELAX-AHF trial, evaluating adults hospitalized due acute heart failure and showed a lower incidence of worsening heart failure symptoms during the hospitalization and in an exploratory analysis, a reduction on mortality rates at 180-days after hospitalization (42). Following this, the RELAX-AHF-2, a placebo controlled trial designed to evaluate the impact on cardiovascular mortality in this inpatient adult population. This study enrolled more than 7,000 patients, comparing a serelaxin infusion for up to 48 h to placebo: unfortunately there was no improvement in mortality, rehospitalization for HF or renal failure incidence at 180 days, or the length of the index hospital stay (43). Meanwhile, in 2014, a phase II clinical trial was also initiated in children to evaluate the pharmacokinetics and safety of serelaxin associated with standard of care therapy in children with acute HF, named the RELAX-PEDS-PK trial. Unfortunately, the pediatric study was terminated by the sponsor after the negative results arising from RELAX-AHF-2.

#### Istaroxime

Istaroxime is an investigational drug initially developed in 2004, and currently in phase II clinical trials in the European Union. It is a first in class synthetic agent, modeled on the structure of digitonin, with both inotropic and lusitropic properties. The mechanism of action is *via* an inhibitory effect on the sarcolemmal Na+/K+ ATPase channel, and a stimulatory effect on sarcoplasmic reticulum Calcium 2+ ATPase isoform 2a (SERCA 2a). The net effect is an increase in intracytoplasmic calcium in systole, and an increase in sarcoplasmic reticulum calcium uptake in diastole. Pharmacologic behavior suggests more inotropic effect and less proarrhythmic effect than digoxin. Originally conceived of as an intravenous drug for acute heart failure syndrome, the HORIZON-HF trial demonstrated a reduction in pulmonary capillary wedge pressure, and improved diastolic function following a 6 h infusion in hospitalized HF patients (44). There is currently no data available regarding pediatric use.

#### Synthetic Natriuretic Peptides

Natriuretic peptides constitute an important physiologic response to volume overload and increased ventricular wall stress, and are a useful serum biomarker of the heart failure response. They have also been explored as therapeutic agents, acting by inducing vasodilation, diuresis, and natriuresis through the natriuretic peptide receptor/particulate guanylate cyclase/cyclic guanosine monophosphate pathway. Synthetic natriuretic peptide analogs are therefore currently being investigated for use in adults with ADHF. Carperitide is a recombinant atrial natriuretic peptide analog which is approved in Japan for the treatment of ADHF (45). Ularitide is a synthetically derived form of urodilatin, an endogenous peptide secreted from the distal convoluted tubule which regulates renal sodium reabsorption and water homeostasis. Early findings in phase II clinical trials ularitide in adults with ADHF suggest a significant reduction in pulmonary capillary wedge pressure (PCWP) and an improvement in dyspnea symptoms (46). In a larger study of 221 AHF patients, ularitide was found to favorably reduce PCWP and stroke volume (SV) in all the three dose groups that were studied (47). A randomized, placebo controlled, phase 3 study (TRUE-AHF) is currently underway in adults with ADHF. No data is available regarding pediatric use (48).

### Chronic Heart Failure

Chronic heart failure in children is a more complex designation, since it implies the presence of structural remodeling of the heart, and may be present (according to the AHA and ACC framework illustrated in Table 3) irrespective of current symptoms. Patients with any prior symptomatic presentation of HF are deemed to have HF class C in this rubric. This does not always apply well to children for whom surgical repair of a VSD, for example, may be curative, or for those who recover from acute myocarditis with no residual disease. In this respect, “*ventricular remodeling*,” which we define here as the adverse structural adaptation of myocardial tissue, with associated changes in ventricular morphology. Remodeling must be present to support a diagnosis of chronic heart failure. For the purposes of this discussion, such symptoms or evidence of ventricular remodeling should be present for a duration of at least 3 months, in order to identify chronic HF, however the point at which acute HF becomes chronic HF remains arbitrary. Although the duration of HF appears to be relevant to survival in adults, this point has not yet been validated in children (50). Symptom severity is typically classified by either the Ross classification in infants and preschool children, or the NYHA classification in older children and adolescents (14).

**Table 3:** Classification of HF accordingly with the American heart association and American college of cardiology (49).

**Heart failure stage**	**AHA/ACC description**
Stage A	Patients without identified structural or functional cardiac abnormality or ventricular function abnormality but at high risk of developing HF because of the presence of a condition strongly associated with the development of HF. Examples: anthracycline exposure, known pathogenic sarcomeric gene mutation including dystrophinopathies.
Stage B	Patients with structural heart disease or ventricular function abnormality that is strongly associated with the development of HF but without HF signs or symptoms, past or present. Examples: asymptomatic patient with CHD status post-surgical correction with residual lesion, isolated left ventricle non-compaction.
Stage C	Patients with current or prior symptoms of HF associated with underlying structural heart disease, or ventricular function abnormality. Examples: acute myocarditis., dilated cardiomyopathy, mitral or aortic regurgitation.
Stage D	Patients with advanced structural heart disease and refractory symptoms of HF requiring specialized interventions. Example: Inotropic dependency patient in end stage of dilated cardiomyopathy.

Most of the recommendations for chronic pediatric HF therapy were extrapolated from adult heart failure trials (5, 9, 14). Treatment therefore includes blockade of the renin-angiotensin-converting enzyme-aldosterone system, as a cornerstone of therapy. Options available include angiotensin converting-enzyme (ACE) inhibitors, or angiotensin receptor blockers (ARBs), combined with a mineralocorticoid receptor antagonists (MRA), either spironolactone or eplerenone, as the current standard of care. The addition of a β-adrenergic receptor antagonist (β-blocker) for both asymptomatic and symptomatic patients with chronic HFrEF is also commonplace, but less well-supported in the pediatric literature. Maintenance diuretic therapy is reserved for patients with intractable volume overload related symptoms, secondary to heart failure. Digoxin is not routinely recommended, but can be useful for patients who remain symptomatic after treatment with the above-stated drugs is maximized. Unfortunately, even using all recommended medications for the condition, the outcome of chronic HF in children is frequently unsatisfactory (51). Given this reality, the potential role of non-traditional or more recently introduced drugs becomes an important consideration.

#### Ivabradine

Ivabradine targets the voltage-regulated inward funny current (I_f_) in sinoatrial tissue, and slows the rate of phase-4 depolarization, reducing heart rate. Following initial approval of this drug for the management of refractory angina in adults in the European Union in 2005, the drug was studied in adult HF populations, in the SHIFT and BEAUTIFUL studies. Although the results were somewhat discrepant, a pooled analysis suggested a decreased risk in HF hospitalization and mortality in patients with HFrEF (52). This resulted in a class IIa indication for the treatment of stable symptomatic HF with LVEF of <35%, and persistent tachycardia on optimal β-blocker therapy, or in patients unable to tolerate β-blockers. The safety of ivabradine in children has been validated in a pediatric phase II/III dose finding clinical trial of children with stable HF (53). In this study, ivabradine resulted in a reduction in heart rate, an increase in EF, and a trend toward improved quality of life. There were no significant differences in NT-pro BNP levels between ivabradine and placebo treatment groups noted. Subsequent published experience using ivabradine in pediatric HF remains limited. A retrospective analysis of a small cohort of Duchenne muscular dystrophy patients with reduced LVEF treated with β-blockers with or without ivabradine suggested an improved LVEF and improved freedom from major adverse cardiac events, using ivabradine in a heart rate reduction strategy (54). Of interest, a recent population-based cohort study of young adult males indicates that heart rate elevation in younger ages is associated with incremental risk of later development of HF in adults (55). Meanwhile, a retrospective analysis of children with dilated cardiomyopathy from the Pediatric Cardiomyopathy Registry found that elevated heart rate was independently associated with death or transplantation, after correcting for age, ventricular function, and cardiac medication use (56). There are a number of reports describing the efficacy of ivabradine in terminating or at least ameliorating inappropriate tachycardia due to atrial automatic tachycardias in children, with durable reduction in heart rate and reversal of heart failure symptoms (57). The United States FDA recently granted approval for the use of ivabradine in children, with a labeled indication for children with symptomatic heart failure >6 months of age.

#### Sacubitril-Valsartan

Sacubitril-Valsartan is a first in class drug, which combines a neprilysin inhibitor, and an angiotensin II receptor antagonist. Neprilysin, the target of sacubitril, is a widely expressed enzyme whose main cardiovascular system effect is to break down natriuretic peptides. With the inhibition of neprilysin, circulating natriuretic peptide levels rise, most notably B-type natriuretic peptide (BNP). The hemodynamic effects of this increase in BNP include vasodilation and diuresis. Neprilysin inhibition also activates the RAAS pathway, and thus the combination of neprilysin with a classical RAAS antagonist is a logical choice of a combination-agent. Initial attempts to develop such a drug combined an ACE inhibitor with sacubitril: this combination drug, omapatrilat, resulted in an excessive amount of angioedema, and was abandoned (58). However, combining the angiotensin receptor blocker (ARB) valsartan with sacubitril has been found to result in significant benefit in the treatment of HFrEF, without an increased risk of angioedema (59).

In the landmark PARADIGM-HF Trial, the angiotensin receptor-neprilysin inhibitor sacubitril/valsartan was shown to be superior to enalapril in reducing the primary endpoint of death or heart failure hospitalizations in adults with HFrEF (60). This was the largest prospective randomized trial of a drug in heart failure ever performed at that time, with over 8,000 participants from over 1,000 sites. There was a highly significant 18% relative risk reduction for death from cardiovascular causes or hospitalization for heart failure, by sacubitril/valsartan compared with enalapril. Sacubitril/valsartan was however associated with a higher incidence of hypotension, although there was a lower incidence of elevated creatinine or serum potassium when compared with enalapril. Subsequent *post-hoc* analyses of the PARADIGM-HF trial have shown that sacubitril/valsartan is superior to enalapril in reducing sudden cardiac death in adults, and has comparable efficacy and safety profiles across all doses of drug, with similar effects in black adults compared with other races (61–63). Sacubitril/valsartan has also been shown to improve quality of life, reduce pulmonary artery pressure, and reduce the biomarker N-terminal proBNP to a greater extent than enalapril in adults HFrEF and ADHF (64–66). Hence, this drug which was also the first class of drug ever developed solely for the treatment of chronic HFrEF has attained a guideline endorsed Class 1 indication for the treatment of HFrEF (67).

Based on the success of the PARADIGM-HF trial, a comparison trial of sacubitril/valsartan vs. enalapril in pediatric patients with a systemic left ventricle with HFrEF and Class C heart failure was proposed. The resultant PANORAMA-HF trial utilized both a unique study design and a unique endpoint for this type of drug trial (68). The study used a two-part platform sequential design, with the first part being a pharmacokinetic/pharmacodynamic (PK/PD) study performed sequentially in three different age groups, starting with the oldest children (6–18 years old), then the next youngest age group (1–6 years old), and finally the youngest (1 month to 1 year). Once the PK/PD data was obtained and analyzed in each age group and the target dose for part 2 of the study confirmed, then the 2nd part of the study sequence was opened for enrollment of patients in that age group: a double-blind, randomized head-to-head trial comparing sacubitril/valsartan, and enalapril. In working with the US FDA, it was decided to accept a global rank endpoint that had been used previously in acute heart failure studies in adults (69).

In the middle of this trial (October 2019), the FDA approved the use of sacubritil/valsartan for pediatric patients with symptomatic heart failure with systemic left ventricular systolic dysfunction, 1 year of age and older. This was based upon the analysis of 110 subjects in the PANORAMA-HF trial, where they analyzed NT-proBNP levels at baseline and 12 weeks into the study. Based on this data, the FDA was able to show comparable reductions in NT-proBNP between enalapril and sacubitril/valsartan. These changes paralleled what was seen in the PARADIGM-HF trial and therefore was felt to be adequate data to infer that sacubritil/valsartan could be approved for use in children ≥1 year. At the time of writing, the PANORAMA-HF trial has completed enrollment and is now waiting for all subjects to complete the 52 weeks of therapy before performing data analysis. Dosing recommendations in younger children are currently pending.

## Newer Drugs in Chronic Heart Failure

### Omecamtiv Mecarbil

The discovery of the first small molecule activator of cardiac myosin, omecamtiv mecarbil, was reported by Morgan et al. in 2010 and has since progressed through to phase III clinical trials in adults with HFrEF (70, 71). This agent is a first-in-class drug, with demonstrated selective ability to increase cardiac myosin ATPase activity. Mechanistically, it binds to the base of the lever-arm of the myosin protein and permits the easier release of ADP-P from the myosin-actin-ADP complex, permitting more myosin heads to be employed in contraction. As a consequence, it increases the contractile force and the duration of systole, with no increase in myocardial oxygen consumption (72).

Evidence of the clinical efficacy of omecamtiv mecarbil is pending, with data already reported from phase I and II studies, and with two phase III studies (GALACTIC HF studying outcomes in HFrEF, and METEORIC-HF studying the impact on exercise capacity, reporting later in 2021). In GALACTIC HF, 8,256 adult inpatients and outpatients with symptomatic chronic HF and an EF of 35% or less were recruited to receive omecamtiv mecarbil or placebo, in addition to standard heart-failure therapy. The primary outcome was a composite of a first heart-failure event (hospitalization or urgent visit for heart failure) or death from cardiovascular causes. This outcome measure was met, with a small reduction in relative risk of 2% (hazard ratio 0.92), and a 10% reduction in NT proBNP levels in the treatment group. The major impact appeared to be in a reduction of HF events rather than in mortality (73). The effect of omecamtiv mecarbil was more pronounced (with a risk reduction of 17%) in patients with a EF of <22%, which suggests a greater therapeutic benefit in the patients with more severe contractile impairment, consistent with the mechanism of action of the drug (74, 75). Some concern has been expressed in that a small elevation of troponin levels was observed in omecamtiv mecarbil treated patients, as was reported in two previous phase II studies (ATOMIC-HF and COSMIC-HF) (75, 76). No data is currently available for children.

### Vericiguat

The cyclic GMP (cGMP) pathway has been implicated as an important regulator of endothelial function in both primary and secondary pulmonary hypertension, and is relevant to myocardial and vascular smooth muscle dysfunction in HF states as well. Vericiguat is a new oral soluble guanylate cyclase stimulator, now approved for the treatment of patients with HFrEF. Its mechanism of action involves the enhancement of the cGMP pathway stimulation and directly increasing endogenous nitric oxide by stabilizing the nitric oxide binding to its site. In the recently reported VICTORIA trial, adult patients with chronic symptomatic HFrEF and an EF of <45% were monitored for a composite endpoint of cardiovascular death or first HF hospitalization (77). The trial demonstrated a reduction in risk of cardiovascular death, all cause death, and HF hospitalization. No data is currently available for children.

### Sodium–Glucose Co-transporter 2 Inhibitors

Dapagliflozin and Empagliflozin are drugs approved for the management of type II diabetes, targeting the sodium-glucose co-transporter (SGLT-2) in the proximal tubule of the kidney. Recent trials in adults have demonstrated a beneficial effect of SGLT-2 inhibition on survival in adult patients with HFrEF with or without type II diabetes mellitus. In DAPA-HF, outpatients with EF <40% and NYHA Class II-IV symptoms showed a 30% reduction in the occurrence of first HF admission and an 18% reduction in cardiovascular death on a 10 mg daily dose of dapagliflozin (78). This result was largely replicated in the EMPEROR-reduced trial using empagliflozin, lending credibility to this previously unsuspected benefit in non-diabetic patients with HFrEF. It showed a reduction in cardiovascular death of 19.4% in the treatment group vs. 24.7% in the placebo group (hazard ratio 0.75) (66). There was also reduction in HF hospitalization. The precise mechanism of SGLT-2 inhibition in achieving this effect is uncertain. Recent experimental work suggests an antiapoptotic effect mediated *via* sarcolemmal sodium-hydrogen co-transporter blockade (79). However, other proposed benefits of the drug are numerous, including diuresis and natriuresis, reduced LV filling pressures and ventricular afterload, improved vascular function, improved myocardial efficiency by permitting ketone-based myocardial metabolism, and reduced oxidative stress and inflammatory cytokine production all proposed (80). No clinical trial or efficacy data is yet available in children.

## Current Practice: Which Drug for Which Patient?

**Figure 2:**
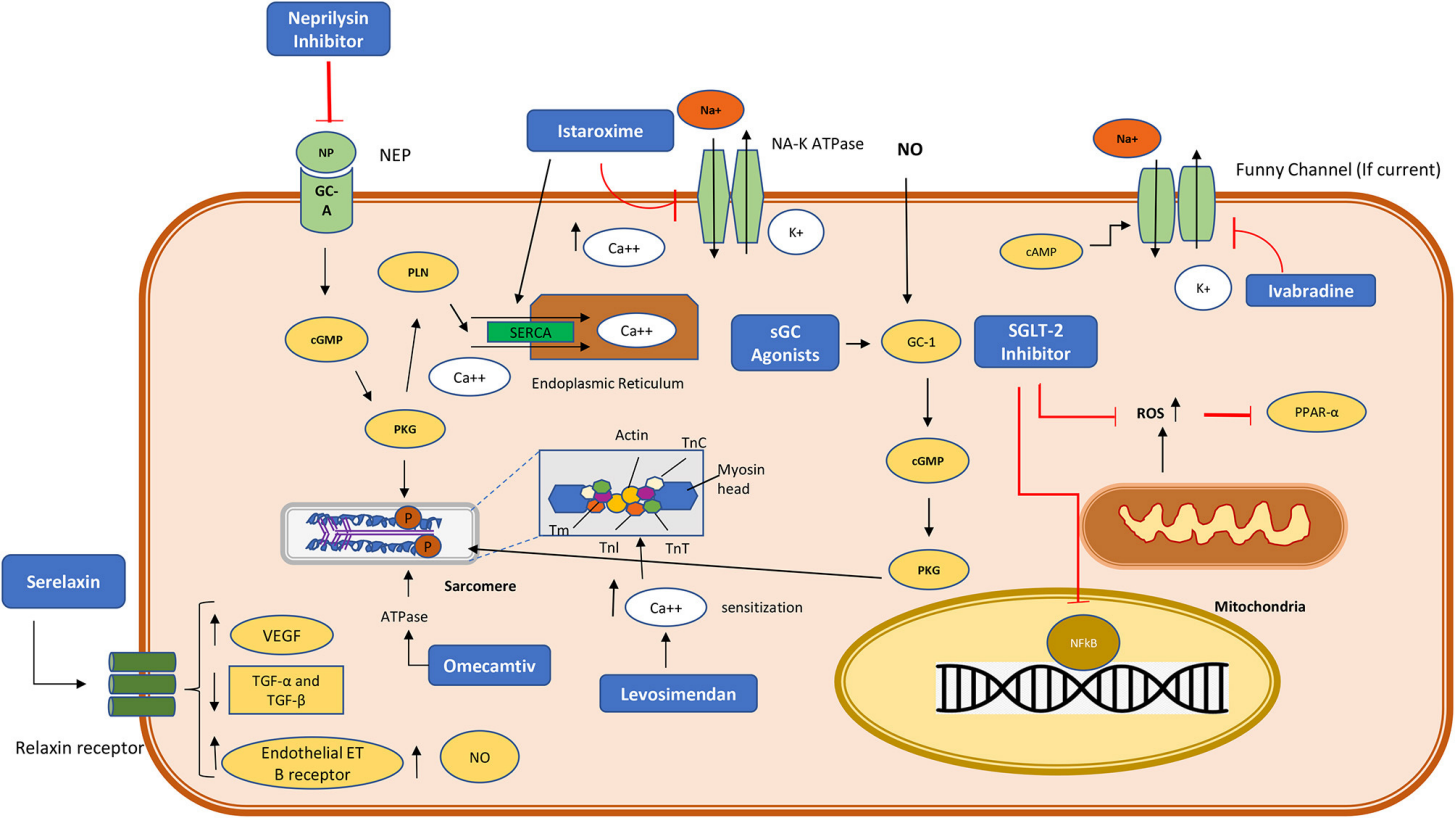
Cardiomyocyte pathway of action of the newer drugs currently used or proposed in heart failure. NP, neprilysin; sGC, soluble guanylate cyclase; cGMP, cyclic guanylate monophosphate; SGLT-2, Sodium glucose co-transporter- 2; P, phosphorylation modification; ROS, reactive oxygen species; PPAR-α, peroxisome proliferator activated receptor alpha; NO, nitric oxide; NFκB, Nuclear factor kappa B transcription factor; cGMP, cyclic guanylate monophosphate; PLN, phospholamban; SERCA, sarcoplasmic/endoplasmic reticulum Ca2+-ATPase; ATP, Adenosine triphosphate; cAMP, cyclic adenosine monophosphate; VEGF, Vascular endothelial growth factor; ET, endothelin receptors; TGF, Transforming growth factor. PKG, protein kinase G; TnI, troponin I; TnC, troponin C; TnT, troponin T; Tm, Tropomyosin.

**Figure 3:**
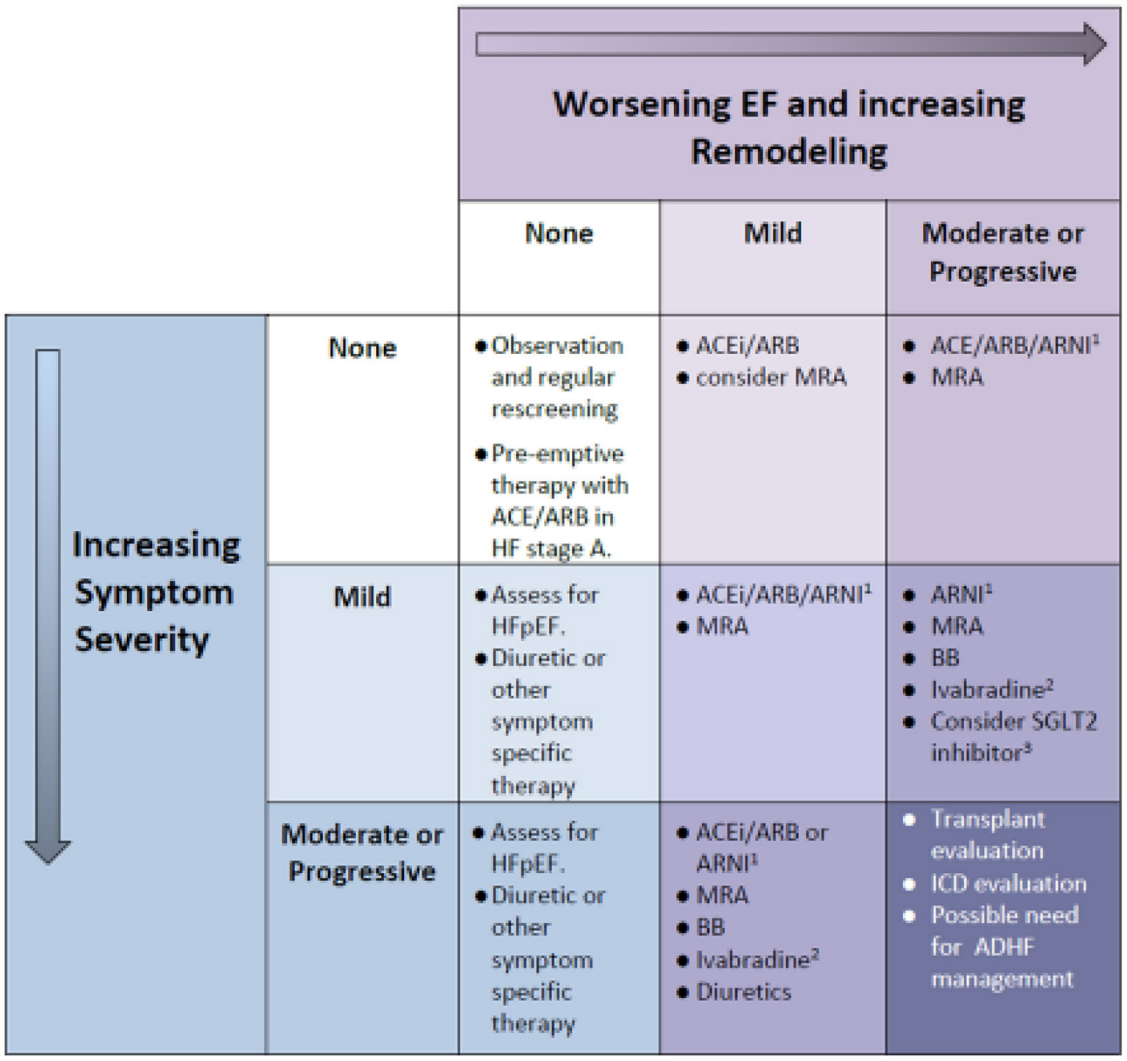
Which Drug for Which Patient: a simplified intuitive guide to the assignment of medical treatment in chronic HF. Remodeling severity is portrayed on the horizontal axis, with increasingly severe symptoms on the vertical axis. Note that progressive symptoms in the absence of LV dilation implies restrictive diastolic function (a form of HFpEF), which may be refractory to management and also require assessment for cardiac transplant and/or ICD. When symptoms are severe and remodeling is advanced (lower right cell) we revert to acute decompensated heart failure management. All patients require an expert assessment and a tailored approach to management depending on the etiology of HF, the severity of decline in EF and degree of LV remodeling (LV dilation). ^1^Indications exist in adults, and use in children is FDA approved, although the outcome of a clinical trial in children is still awaited. ^2^Indication is based on a phase II clinical trial in children, with FDA approval given for children over 6 m age. ^3^Class IIa indication in adults with HFrEF. ACE, angiotensin-converting enzyme inhibitor; ARNI, angiotensin receptor blocker/neprilysin inhibitor; BB, beta blocker; HFpEF, heart failure with preserved EF; MRA, mineralocorticoid receptor antagonist; SGLT-2, sodium glucose co-transporter protein 2.

Given a wider variety of current or potential therapies for both acute and chronic HF, pediatric cardiologists and intensive care specialists find themselves in a period of transition, with limited guidance as to the applicability of specific drug therapies for children, and with more drugs introduced, or likely to be introduced soon. As we have suggested, the optimal approach to acute decompensated heart failure is to recruit systolic function and adequate blood pressure to achieve decongestion (diuresis) and reduce lactate production at first. Thereafter we recommend graduated withdrawal of pure inotropic support (epinephrine or dopamine) first, followed by reduction in dosage of inotropic vasodilators (such as milrinone) over 48–72 h, coupled with the introduction of sacubitril/valsartan or an ACE inhibitor as maintenance therapy. As a general guide, the authors still endorse a phased approach of introducing a combination of oral maintenance therapy drugs following de-escalation of vasoactive drugs in patients with ADHF. The logical approach remains to initiate RAAS pathway inhibition first, as well as an MRA drug. There is now evidence that direct introduction of sacubitril/valsartan is tolerated well in this setting, and so this may become the preferred option (66). This can be followed by a β-blocker being commenced prior to hospital discharge. Target dosing of RAAS inhibitors can be achieved within 7 days in most patients, but β-blockade typically takes longer (81). Additional treatments such as ivabradine will be indicated on some patients who are unable to attain a satisfactory reduction in heart rate on the above approach. The indications for an SGLT2 inhibitor are uncertain at this time, but since the drug has already been adopted in adults with HFrEF, it is not unreasonable to use this in older adolescent patients with HFrEF due to DCM, who have persistent symptoms or progressive remodeling. The myocyte pathway of action of traditional and newly proposed therapies is indicated in Figure 2, and an intuitive approach to selecting drug therapy for specific patient groups is provided in Figure 3. This approach considers the severity indicators of symptoms of HF, and evidence of LV remodeling as key indicators, since they are strongly associated with risk of death or transplantation in HFrEF in several studies in children (82).

## Serum Biomarkers as a Guide to Management of Pediatric HF

Serum biomarkers have acquired an important role in heart failure management. From initially being purely a diagnostic tool, they have since been used to monitor treatment response, and have also become an acknowledged surrogate endpoint in Pediatric and Adult HF clinical trials. BNP is the best studied of these, and yet for adults with HFrEF the data is conflicting. Biomarker-directed care, while demonstrated to be helpful in smaller trials has however not been validated in a large-scale randomized trial in adults, or in any meta-analysis of smaller studies (83, 84). In the pediatric population, the use of BNP and or NT proBNP has been recommended to stratify the severity, and to monitor the progression of HF. To date there is some evidence in children that BNP or NT-proBNP can act as surrogate markers of the likelihood of admission to hospital, as well, the necessity of mechanical circulatory support, or heart transplant after admission with ADHF (85–88). Recently, a multi-biomarker approach to risk stratification in heart failure has become popular. This is exemplified by point-assessments of risk for death or adverse outcome in adults with non-ischemic cardiomyopathy using assessment tools like the MAGGIC scale, and others (89). In children the Pediatric Cardiomyopathy registry has published preliminary results suggesting added value in incorporating a panel of serum biomarkers in the initial risk assessment of children presenting with heart failure (90).

## Conclusions

The most recent published guidelines from the United States and Canada give recommendations for the management of HF in children provide recommendations for the management of acute HF including diuretics for fluid overload, judicious use of inotropes for hemodynamic instability, and ACE inhibitors, (ARBs if intolerant to ACE inhibitors), β-blockers, and MRAs for chronic HF. However, new guidance is required for the indications and timing of ivabradine and sacubritil/valsartan, and potentially for the future adoption of the other drugs indicated here, into the management of pediatric HF.

Until very recently, medications made available for the treatment of HF have traditionally been developed and tested in adults with HFrEF, and gone on to be used in children, with or without clear experimental evidence of efficacy. The American Academy of Pediatrics has stated that “It is morally imperative to formally study drugs in children so that they can enjoy appropriate access to new and existing therapeutic agents” (91). Although pediatricians are in agreement with this, there are many barriers to performing rigorous drug trials in children. These barriers include patient recruitment challenges, a need for innovative study design, regulatory and financial barriers. Since the 1997 U.S. Food and Drug Administration Modernization Act, pharmaceutical companies have been motivated to perform drug trials in children in order to obtain 6 months of patent exclusivity for performing these trials in children. In the European Union, a Pediatric Investigation Plan is a requirement for new drug licensing. These measures have provided significant incentive to proceed with drug trials in children, however challenges still remain. The ability to conduct successful trials in children with HF has been demonstrated, and should be encouraged by regulators and pharmaceutical developers alike.

## Author Contributions

All authors contributed to the conceptual design, data review, writing, and review of this manuscript.

## Conflict of Interest

RES is a consultant for Novartis, Bayer, and Bristol Myers Squibb. PFK is a consultant for Novartis. The remaining author declares that the research was conducted in the absence of any commercial or financial relationships that could be construed as a potential conflict of interest.

## Publisher's Note

All claims expressed in this article are solely those of the authors and do not necessarily represent those of their affiliated organizations, or those of the publisher, the editors and the reviewers. Any product that may be evaluated in this article, or claim that may be made by its manufacturer, is not guaranteed or endorsed by the publisher.
